# DNA ligase IV dificiency with elevated serum IgG levels suspected to have myelodysplastic syndrome: a case report

**DOI:** 10.1186/s12887-022-03655-x

**Published:** 2022-10-11

**Authors:** Miao Huang, Guoqing Dong, Xiyan Lu, Fei Xiao, Qixin Zhou, Shaoyi Zhang

**Affiliations:** Department of Pediatrics, Shenzhen Maternity and Child Healthcare Hospital, Shenzhen, China

**Keywords:** LIG4 dificiency, Immunodeficiency, Elevated IgG, Case report, Myelodysplastic syndrome

## Abstract

**Background:**

Ligase IV (LIG4) dificiency is a very rare clinical syndrome with around 50 cases reported to date. This syndrome is caused by biallelic pathogenic variants in the LIG4 gene, which cause DNA damage repair disorders, mainly manifesting as severe immunodeficiency.

**Case presentation:**

We report the case of a 15-month-old male child with pancytopenia, growth retardation, microcephaly, history of vaccine-related rubella, elevated immunoglobulin G, and decreased T- and B lymphocytes. Next-generation sequencing revealed LIG4 pathogenic genes and compound heterozygous mutations, namely the missense mutation c.833G > T (p.Arg278Leu) and deletion mutation c.1271_1275del (p.Lys424Argfs*20).

**Conclusion:**

This case suggests that LIG4 dificiency can manifest not only as immunodeficiency but also with increased serum IgG levels and pancytopenia, which constitutes an additional clinical phenotype. Furthermore, this case suggests that LIG4 deficiency should be considered upon differential diagnosis of myelodysplastic syndrome in children.

## Background

LIG4 dificiency is a very rare genetic disease that is caused by mutations in the LIG4 gene (13q22-q34). It is associated with impaired DNA double-strand break repair mechanisms, and its incidence is currently unknown [1]. Clinical manifestations mainly include microcephaly, abnormal facial features, growth and developmental delays, skin abnormalities, pancytopenia, and severe combined immunodeficiency [2–4]. The clinical phenotype of LIG4 dificiency is related to the location and type of mutation, with great heterogeneity in the clinical manifestations of patients [5, 6]. The characteristics of LIG4 dificiency in a Chinese cohort mainly comprised missense mutations (c.833G > T, p.R278L) and frameshift mutations caused by deletions c.1271_1275delAAAGA (p.K424Rfs*20) [7]. Furthermore, growth restriction, overt microcephaly, combined immunodeficiency, and low IgG levels have also been observed.

Pancytopenia is common in hematological diseases and secondary to other diseases. The most common causes are suppression of bone marrow hematopoiesis, or malignant hematological diseases of the bone marrow hematopoiesis (MDS), or immunological disease. MDS is a disorder characterised by ineffective haematopoiesis, which has a tendency to evolve into acute myeloid leukaemia (AML) [8]. Primary immunodeficiency disease (PID) can lead to haematological abnormalities, manifesting as pancytopenia, and haematological malignancies. In 2020, international union of immunological societies identified bone marrow failure as a new class of PID. These mainly include bone marrow failure syndrome, dyskeratosis congenita, and Fanconi anemia. Therefore, to improve our understanding of the clinical manifestations of LIG4 dificiengcy, the basic conditions of an encountered patient are described here.

## Case presentation

The patient was a 15-month-old boy who was admitted to our hospital due to failure to growth and poor appetite. He was a full-term gestational child delivered at 40 + 3 weeks of pregnancy. His birth weight was 2.3 kg, head circumference was 31 cm, and body length was 50 cm. There was no history of neonatal asphyxia. He resistance to complementary foods. At 6 months of age, he underwent an operation for polydactylism. He had repeated eczema-like rashes and scattered bleeding points all over the body. He did not have repeated respiratory or digestive tract infections after birth. Moreover, he had no family history of malignant tumours. Regarding his vaccination, he completed the BCG dose, third dose of hepatitis B vaccine, third dose of polio vaccine, third dose of DPT vaccine, second dose of meningococcal polysaccharide vaccine (Group A), and first dose of measles vaccine. The patient had not received any vaccination in the last 5 months before admission.

**Fig. 1:**
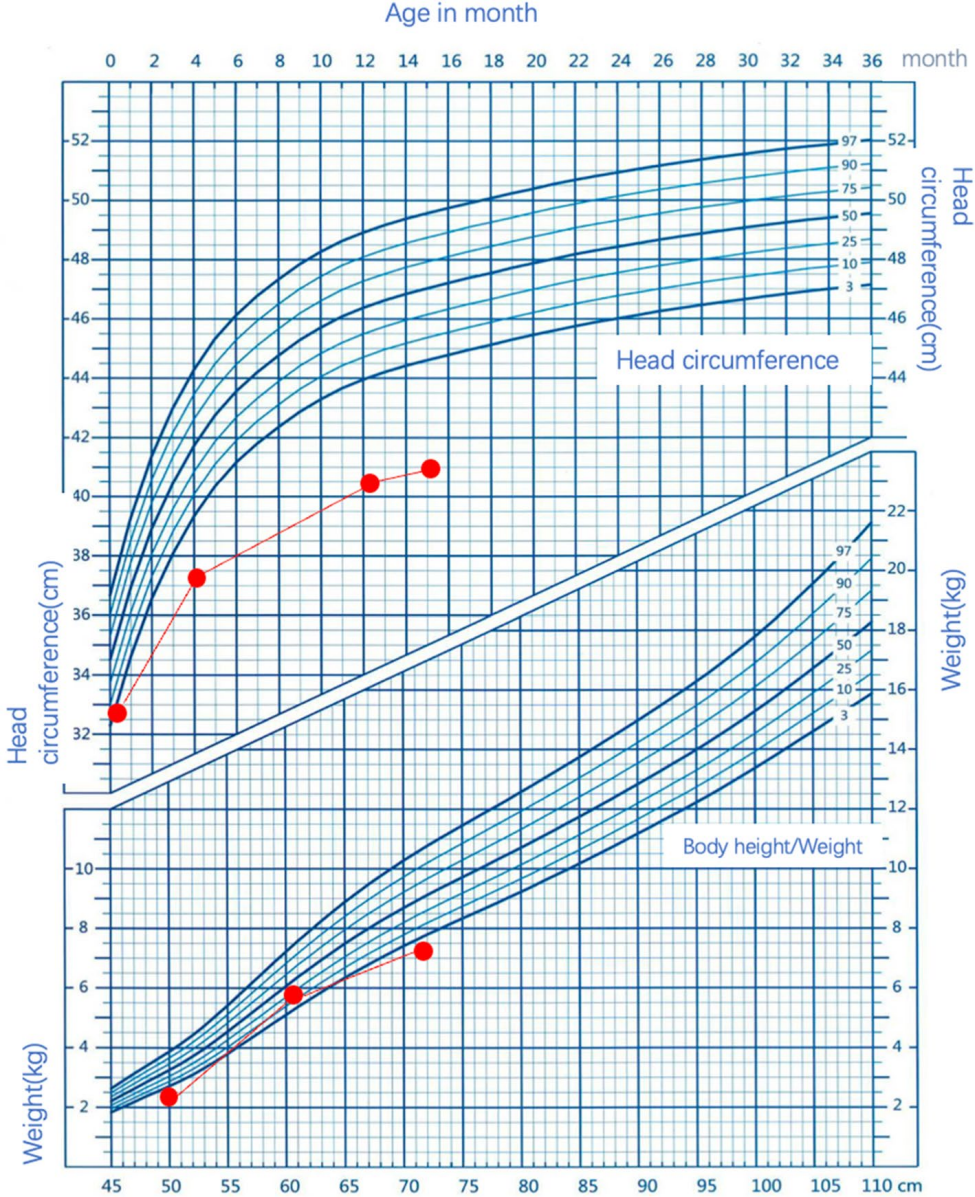
0-3 years old head circumference, growth percentiles for boys in China

His physical examination showed a weight of 7.4 kg (− 3 SD), height of 72 cm (− 2 SD), head circumference of 40.2 cm (− 3 SD, microcephaly), six primary teeth, and normal facies (Fig. 1. 0-3 years old head circumference, growth percentiles for boys in China). The patient’s psychomotor development was normal. The liver and spleen were not enlarged. The full blood count revealed a low white blood cell count (WBC) (2.41 × 10^9^), neutrophil count (NE) (1.40 × 10^9^/L), haemoglobin (HGB) (87 g/L), and platelet (PLT) count (7 × 10^9^/L).

His bone marrow smear showed active bone marrow hyperplasia, granulocyte hyperplasia and maturity disorder, and the proportion of lymphocytes decreased; Erythrocyte proliferation was active; Megakaryocytes proliferate actively, and the proportion of megakaryocytes producing plates was low.

Prior to gamma globulin infusion, immunoglobulin test results revealed an elevated IgG level (18.7 g/L, 4.7–12.3), but normal IgA and IgM values. Upon re-checking 2 months later, IgG was still elevated (22.1 g/L, 4.7–12.3), whereas IgG4 protein quantification was normal (0.477 g/L，0.08-1.4). Lymphocyte immunophenotyping was performed twice, which showed that the counts of T lymphocytes and especially CD4 + T cells were significantly diminished (Table 1. The patient’s immune function test results). The twice test was performed after corticosteroid treatment.

**Table 1:** The patient’s immune function test results

Item	Age 1 year	Age 1 year 3 months	Normal reference	Characterisation
T%	33.0	60	65.0–79.0	↓
		716/μL	723–2737/μL	
CD4 + T%	15.1	7.54	34–52	↓
		76/μL	404–1621/μL	
CD8 + T%	20.7	49.1	21.0–39.0	↓
		1324/μL	220–1129/μL	↑
B%	11.2	4.02	8.3–18.9	–
		44/μL	80–616/μL	
NK%	11.3	26.8	4.6–26.0	–
		731/μL	84–724/μL	
CD4 + T/CD8+ ratio	0.7	0.15	0.9–2.0	↓
IgG (g/L)	18.7	22	4.7–12.3	↑
IgA (g/L)	1.38	1.57	0.21–1.45	–
IgM (g/L)	0.27	0.30	0.41–1.75	–

The aetiology antibody testing included testing for rubella virus, cytomegalovirus, toxoplasma, herpes simplex virus, human parvovirus B19, and Epstein-Barr virus. This patient was found to be positive for rubella virus antibody IgG and IgM and cytomegalovirus antibody CMV-IgG, whereas he was negative for the other viral IgG and IgM antibodies.

Based on the serological findings, rubella virus was diagnosed. We performed an ophthalmic fundus examination and found that rubella virus retinal vasculitis was visible in both eyes. The manifestations were as follows: no obvious opacity of the lens was observed, and the optic disc was clearly demarcated, orange in colour, without obvious bulge, and had a C/D ratio of approximately 0.3. The colour of retina was relatively dark; white punctate exudation foci were scattered at many places, and no obvious haemorrhage was observed. The retinal artery was small and slightly tonic, with a visible white sheath wrapping on the lateral side of some arteries and foveal reflex. The 30-cm red globe test was positive upon visual screening. Manifestations related to rubella virus infection of the patient included microcephaly, low body weight, and polydactyly, but his intellectual, behavioural, and motor development were normal.

**Fig. 2:**
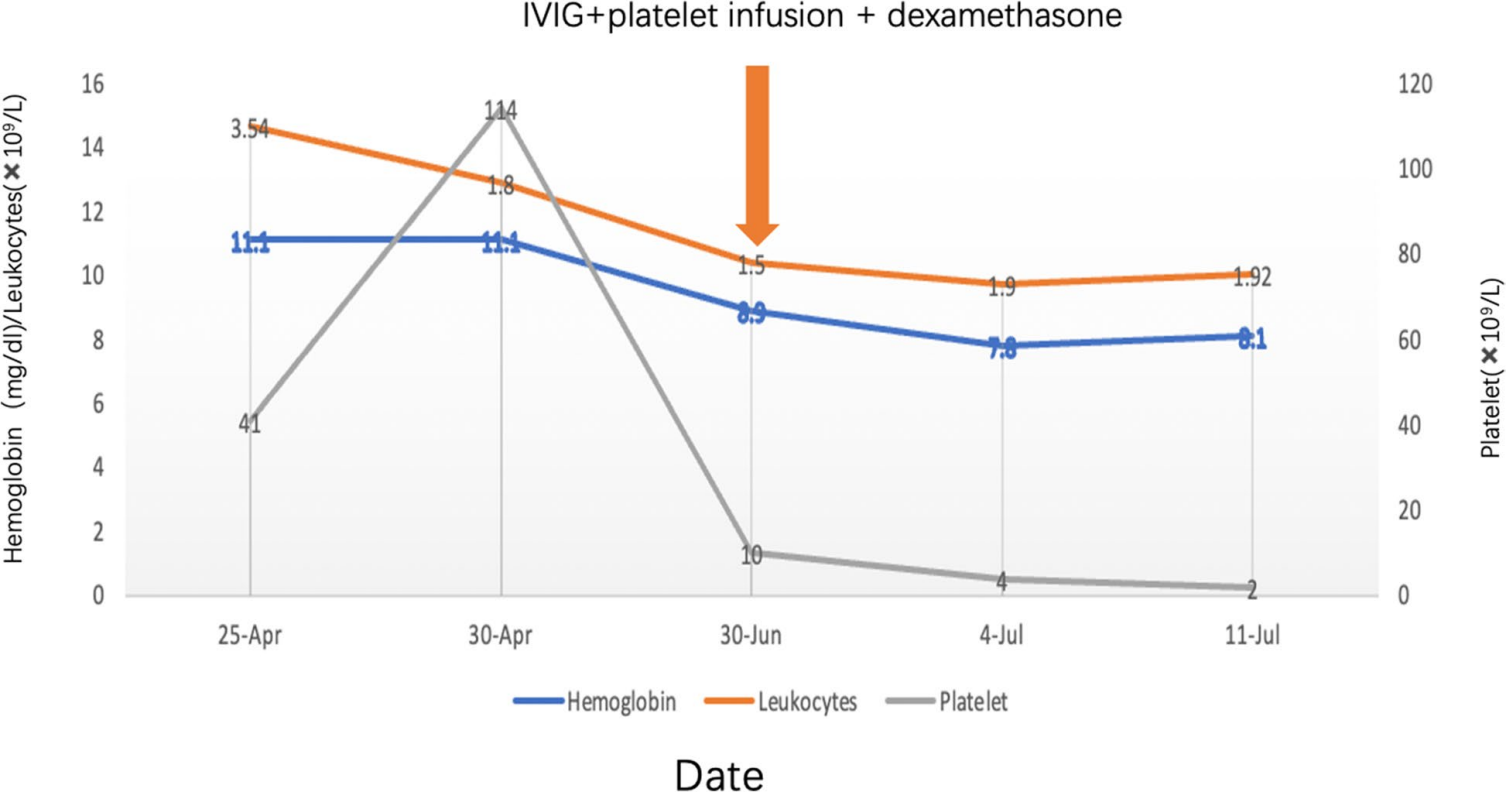
The platelets, leukocytes, and hemoglobin trend during the hospitalization of the patient

Initial management consisted of intravenous infusion of gamma globulin (1 g/kg, three doses), PLT (2 U), dexamethasone (0.2 mg/kg for 4 days), and oral prednisone acetate (0.2 mg/kg for 3 days, and 0.1 mg/kg for 1 week) to suppress the autoimmune reaction. Despite this, the patient’s PLT count did not increase (Fig. 2. The platelets, leukocytes, and hemoglobin trend during the hospitalization of the patient).

Autoimmune disease-related antibody tests, including the direct anti-human globulin test, were positive (4+). PLT-specific and histocompatibility (HLA) antibodies were also positive. Furthermore, his thyroid-stimulating hormone level was high, and thyroxine was normal.

**Fig. 3:**
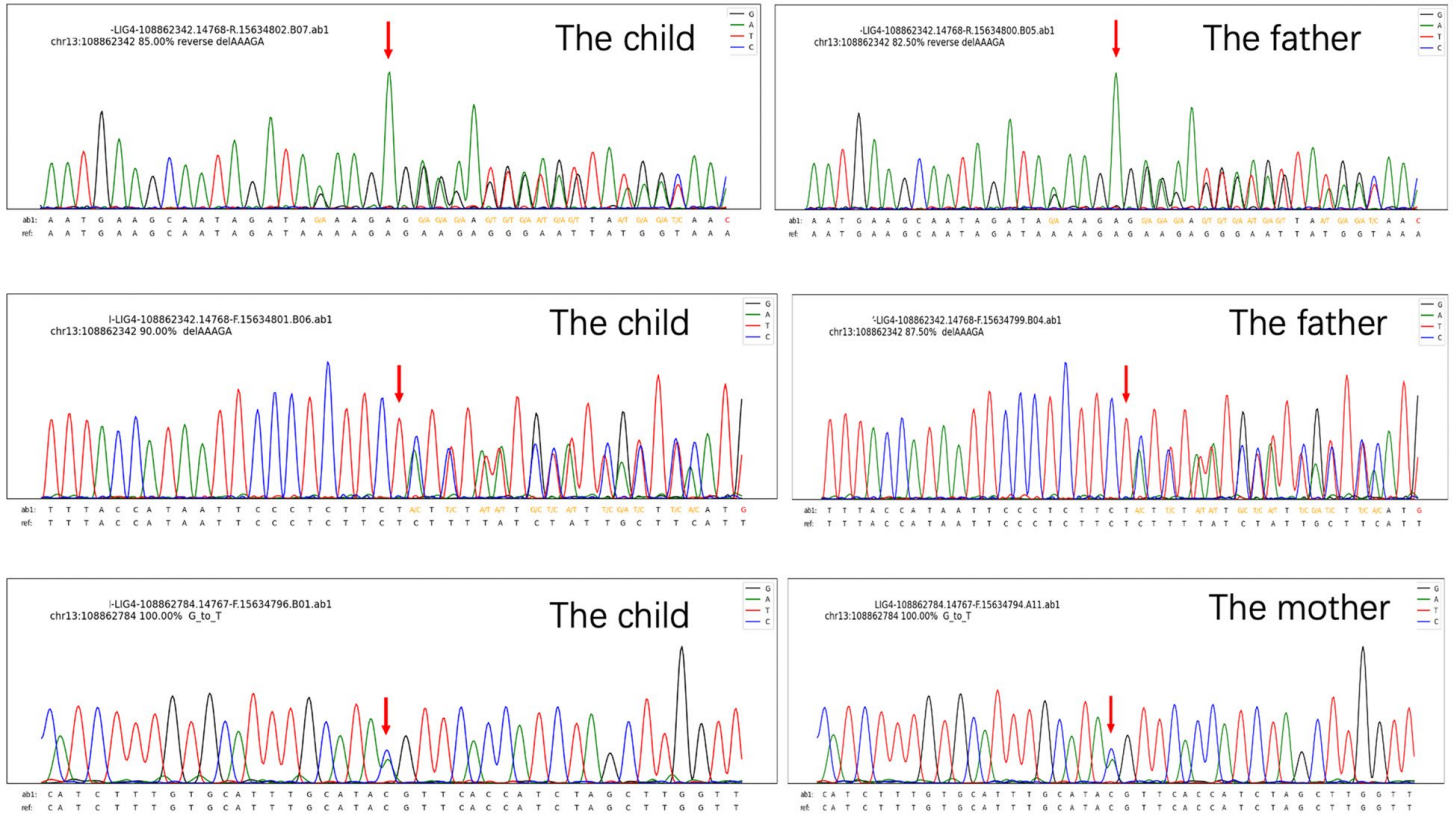
Sequencing results of LIG4 gene of the child and his parents

Whole exome sequencing revealed two heterozygous variants of the LIG4 gene: c.833G > T (p.R278L) and c.1271_1275del (p.K424Rfs*20). Family verification results showed that these two variants were inherited from the mother and father respectively (both heterozygous) (Fig. 3. Sequencing results of LIG4 gene of the child and his parents). According to the ACMG guidelines [9], the pathogenicity classification of these two mutations were likely pathogenic.

The child’s main clinical manifestations were full-term small for gestational age, microcephaly, polydactyly, growth retardation, immunodeficiency, and pancytopenia. Combined with gene sequencing results, the diagnosis of DNA ligase IV deficiency was established. Following the diagnosis of DNA LIG4 dificiency, we stopped all drug treatments for the patient. Notably, he still displayed poor appetite after being discharged from the hospital. Low WBC, NE, HGB, and PLT were also found at the 3-month and 6-month follow-ups, especially PLT, with a minimum value of 7 × 10^9^/L.

## Discussion and conclusions

The clinical phenotypic spectrum of LIG4 dificiency is diverse. In 2018, ltmann et al. [4] reviewed 41 cases of LIG4 dificiency and found that the age of diagnosis ranged from 2 to 17.5 years, and 68% of the patients were women. Most of these patients had microcephaly (80%), growth retardation (70%), radiosensitivity (86%), hypogammaglobulinemia (83%), and extremely low B cell counts (80%). Furthermore, a few cases of LIG4 syndrome were accompanied with bone marrow failure, facial deformity, malignant tumours, psoriasis and other skin abnormalities, congenital dysplasia of the hip, hypogonadism, and sclerosing cholangitis. In 2019, Taskiran et al. [10] reported the first case of LIG4 dificiency with a phenotype of Behcet’s disease combined with dysembryoplastic neuroepithelial tumour. In 2020, Madhu et al. [11] reported that LIG4 dificiency could present with Fanconi anaemia and renal malformations. In our case report, the patient showed pancytopenia, microcephaly, growth retardation, and decreased T- and B lymphocytes. Since whole genome sequencing unveiled a compound heterozygous variant of the LIG4 gene, the diagnosis of LIG4 dificiency was clear. However, there were still some peculiarities in this case.

Regarding the assessment of cellular immunity in the case, we considered the information on low lymphocyte counts to be indicative of PID. It was deduced that rubella infection was the likely result of the poor cellular responses. Additional tests shuold been performed, such as naïve/memory T and B cell test, lymphocyte clonality, lymphocyte proliferation assays, radiosensitivity assays. The CD8+ percentage was elevated the second time (Table 1), which may be related to the administration of dexamethasone and prednisone in the early management of the patient.

Compound heterozygous mutations account for over 90% of patients with LIG4 dificiency, which was the most common gene mutations included c.1271-1275del (Lys424Argfs*20) and c.2440C > T (p.Arg814*). Dard et al. [12] analysed the relationship between immunoglobulin deficiency and genotype in LIG4 dificiency. They found that Lys424Argfs*20 was associated with early symptomatic immunodeficiency and major IgA deficiency, whereas p.Arg814* was associated with major IgM deficiency. Studies have also reported that C.833G > T (p.R278L) and c.1271-1275del (p. K424RFS*20) were common mutation types in the Chinese population [2, 7, 13]. These variants result in milder malformations, with significantly increased IgM and IgA levels and normal or decreased IgG levels. However, this case report is unique, since the immunoglobulin levels and genotypes were not consistent with previously reported cases, suggesting that IgG may be elevated in LIG4 syndrome. As this patient has no history of repeated gastrointestinal or respiratory infections, this may have been related to his increased IgG levels. The patient’s antinuclear and anti-U1-snRNP antibodies were significantly elevated with high thyroid-stimulating hormone levels, we speculated that his elevated IgG level may have also been related to a combination of autoimmunity.

Notably, our patient had a rubella virus infection and was vaccinated at 8 months of age. His mother was not found to have rubella virus infection during pragnancy. This is shares similarities with another case reported by Japanese researchers was vaccine-derived rubella virus infection in a Japanese patient with DNA ligase IV deficiency, whose successful bone marrow transplantation and immune reconstitution led to the eradication of the rubella virus [14]. The vaccine related rebella infection was most probably linked to the patient’s cellular immune defect.

Elevated IgG levels in a PID case warrant further serological work up, mainly regarding oligoclonality and antigen specificity. Oligoclonal increases of serum IgG levels may be encountered in SCID. The patient lacked IgG subclasses, and only found that elevated IgG levels and the IgG4 level was normal. There may be abnormal distribution of IgG subclasses in autoimmune diseases and tumor diseases. It was reliable that information about IgG levels and responses to CMV and rubella,and the same time which is indicative of the ability to develop antigen specific humoral responses. However, there was no information on the patient’s ability to mount antibody responses to a simple vaccination, such as tetanus toxoid, and to weaker antigens, including those of bacterial polysaccharides such as anti-pneumococcal and anti-haemophilus influenza type b antibodies.

Previous studies have also reported improvement of PLT counts after intravenous immunoglobulin injection [7]. However, in this case, the PLT count remained unchanged (< 10 × 10^9^/L) even after treatment with high-dose intravenous infusion of gamma globulin, PLT transfusion, and glucocorticoids. This suggests that conventional immunotherapy was ineffective in our case for the treatment of thrombocytopenia,sendering haematopoietic stem cell transplantation may be the successful treatment [10, 15–17].

Our patient had an initial MDS diagnosis based on persistent pancytopenia. However, there were no obvious signs of ineffective haematopoiesis in bone marrow smear of the patient. The inclusion criteria of suspected paediatric MDS are as follows, including any of the following cytopenias: low WBC, low HGB, low PLT < 150 × 109/L, hypocellular bone marrow [18]. Paediatric MDS needs to be distinguished from aplastic anaemia, which can manifest as nucleated red blood cells in peripheral blood and morbid haematopoiesis in the bone marrow. It should also be distinguished from pancytopenia caused by autoantibodies. Glucocorticoids and IVIG infusion may be effective. MDS has phenotypic complexity and extensive genetic heterogeneity [19]. The ineffective haematopoiesis observed in MDS can result in leukaemia. Both PID and MDS have the risk of easily developing haematological tumours, which are closely related to autoimmune diseases. PID often manifests as repeated infection, susceptibility to haematological system tumours, and autoimmune diseases. Additional findings of the patient that could indicate an inherited syndrome and especially PID were chronic rubella virus infection, growth retardation, repeated rash, immunodeficiency,microcephaly and pancytopenia.

In conclusion,we report a LIG4 deficiency which reveals the complexity of this syndrome. It can present with increased IgG levels and pancytopenia, misleading diagnosis towards MDS.DNA LIG4 dificiency is a rare and complex disease. It can present as immunodeficiency, with increased IgG levels and pancytopenia. As conventional immunotherapy for thrombocytopenia may be ineffective. We should be taken to not exclude LIG4 dificiency in patients with MDS, especially in the presence of malformations or signs of immune deficiency.

## Data Availability

All data and material collected during this study are available from the corresponding author upon reasonable request. The DNA sequencing data presented in the study are available in the [the Genome Variation Map (GVM) in Big Data Center, Beijing Institute of Genomics (BIG), Chinese Academy of Science] repository, [https://bigd.big.ac.cn/gvm/getProjectDetail?Project=GVM000381].

## Abbreviations

LIG4: Ligase IV
MDS: Myelodysplastic syndrome
PID: Primary immunodeficiency disease
AML: Acute myeloid leukaemia
PLT: Platelet count
WBC: White blood cell count
NE: Neutrophils
SCID: Severe combined immunodeficiency
